# Requirements and Concerns of Individuals Remitted From Depression for an Early Relapse Detection mHealth App: Focus Group Study

**DOI:** 10.2196/67141

**Published:** 2025-10-23

**Authors:** Tina Coenen, Matthias Maerevoet, Stephanie Chen, Mathias De Brouwer, Sofie Van Hoecke, Ernst HW Koster, Mariek MP Vanden Abeele, Klaas Bombeke

**Affiliations:** 1 imec-mict-UGent Department of Communication Sciences Ghent University Ghent Belgium; 2 Department of Experimental Clinical and Health Psychology Ghent University Ghent Belgium; 3 IDLab Ghent University – imec Ghent Belgium

**Keywords:** mHealth, mobile health, apps, mental health, depression, relapse prevention, self-monitoring, ecological momentary assessment, passive monitoring, smartphone, mobile phone, wearable devices, qualitative research, thematic analysis

## Abstract

**Background:**

Major depressive disorder is often a recurrent condition, with a high risk of relapse for individuals remitted from depression. Early detection of relapse is critical to improve clinical outcomes. Mobile health (mHealth) technologies offer new opportunities for real-time monitoring and prevention of relapse, if the user requirements of the target population are effectively implemented.

**Objective:**

This study investigated the requirements and concerns of individuals remitted from depression for an mHealth app aimed at monitoring depressive symptoms and detecting early signs of relapse through integrating both active ecological momentary assessment data and passive data from the user’s smartphone and smartwatch.

**Methods:**

Three focus group discussions were conducted with 17 participants remitted from depression. Before the focus group, participants had gained some experience with an in-house designed ecological momentary assessment monitoring app, prompting questions regarding their mood multiple times throughout the day. During the focus groups, feedback and insights were gathered on participants’ expectations, requirements, concerns, and attitudes toward a depression monitoring app. A thematic analysis was performed to identify recurring themes and subthemes, shedding light on the desired user experience and functionalities.

**Results:**

We identified 5 main themes. Participants highlighted (1) a need for customization settings, particularly in terms of data collection and sharing, and frequency of self-assessments. They also valued (2) positivity in the app’s design through positive reinforcement and journaling features. Additionally, participants emphasized (3) interventions to be the main motivator for adoption and long-term usage. More specifically, they wanted the app to foster self-awareness, self-reflection, and insights, and to offer support during deteriorations in mental health. Furthermore, participants deemed (4) transparency in data use and machine learning predictions to be essential for building trust. Participants required these functionalities to bear (5) the user burdens of self-monitoring. Key concerns were for passive monitoring to cause a privacy burden and for active monitoring to raise an emotional burden.

**Conclusions:**

Considering the vulnerability of potential users, the design of an mHealth app for early depression relapse detection should be guided by user preferences and approached with caution. Requirements for customization, positivity, interventions, and transparency must be addressed, while minimizing both the emotional and privacy burden. Future iterations should implement these findings to improve and test the app’s acceptability, adoption, and usability for clinical use.

## Introduction

### Background

Major depressive disorder is a debilitating condition that is associated with tremendous personal suffering and substantial societal costs [1,2]. It is the most prevalent of all mental disorders [3]. Moreover, its prevalence is increasing over time [4], and it is often a recurrent or even chronic disorder [5]. Despite the availability of pharmacological and behavioral treatments that can be quite effective in the acute phase, the risk of relapse remains high for individuals remitted from depression [6-8], with depressive remission being defined as the asymptomatic state after a depressive episode [9].

A contributing factor to the high relapse rates is the presence of both a treatment gap [10] and lag [11]. That is, many patients—even those who have previously sought treatment for depression [12]—only seek help when depressive symptoms become severe [13] or persist for a longer period [14]. Provided the reality of waiting lists, this often means that they only receive mental health care when a full-blown depressive episode has unfolded, and intensive treatment is required. Hence, delayed treatment is harmful to patients [15-17]. Recovery rates decline while the risk of persistence increases if patients do not start treatment within the first few months after the manifestation of an episode [18]. Should patients become aware of the re-emergence of depressive symptoms sooner and, therefore, seek help at an earlier stage, symptoms are less likely to escalate. Early detection and intervention are therefore paramount. Scholars and practitioners have broadened their focus accordingly to preventing the onset of depression, especially among individuals remitted from depression [19-23]. Of key importance to such prevention is predicting if and when there will be a novel depressive episode, in which case patients should be notified and encouraged to seek help early on.

In this realm, recent innovations in mobile health (mHealth), that is, the application of mobile technologies for health care services [24], are promising for personalized mental health monitoring. The ubiquity of affordable smartphones and—to a lesser extent—smartwatches makes mHealth increasingly accessible to patients. mHealth technology also provides opportunities for empowerment by fostering self-management. mHealth apps might have less stigma associated with them, lowering the barrier for adoption. Furthermore, most review papers conclude that mHealth interventions are promising in terms of cost-effectiveness as well, especially for depression management [25-29]. The current landscape of depression management apps largely consists of functionalities providing therapeutic support, intervention, treatment, and psychoeducation, followed by tracking of mood, behavior, or thoughts and symptom management [30-32].

In contrast to the myriad of therapeutic apps currently available in the marketplace, this study focuses on an mHealth app for prevention through monitoring depressive symptoms. More specifically, it is about a future app that captures and feeds both active and passive data sources into a personalized supervised machine learning model, being trained for just-in-time detection of early warning signs that are associated with depression relapse. Active data is gathered through ecological momentary assessment (EMA) questionnaires that participants can answer on their smartphones. EMA is a monitoring strategy to assess users’ feelings, behavior, and context in real time in natural settings, thus maximizing ecological validity while avoiding retrospective recall [33,34]. Passive data is sourced from the user’s smartphone and smartwatch. Smartphone data contain phone usage patterns (eg, screen time per app), revealing a user’s digital footprint. Additionally, smartphones include a multitude of sensors (eg, accelerometers, GPS location sensors, and light and proximity sensors), providing detailed measurements about users’ daily life and their environment. Similarly, smartwatches capture physiological, behavioral, and contextual data (eg, heart rate and step count) in a noninvasive and continuous way.

Passive data tracking via these mobile devices is increasingly relevant for digital phenotyping in mental health care. Digital phenotyping is defined as the “moment-by-moment quantification of the individual-level human phenotype in-situ using data from smartphones and other personal digital devices” [35]. The promise of digital phenotyping through mobile devices is in its unprecedented opportunity to objectively and continuously measure an individual’s real-world functioning. It offers novel ways of understanding and measuring disease manifestations, previously reliant on subjective or self-reported data [36]. More specifically, it could be a fruitful approach to identifying symptoms and early signals of depression relapse [37,38]. Furthermore, recent technological advancements in predictive algorithms, machine learning techniques, and artificial intelligence can facilitate making sense of the unprecedented amounts of granular passive data [39] and pave the way toward personalized prevention for depression [40,41]. For example, the relationship between disturbed sleep and depression is well-established [42-44]. Hence, digital phenotyping by tracking changes in sleep patterns via a smartphone or smartwatch may indicate an impending depressive episode [45].

Smartphones are especially well-suited for digital phenotyping due to their worldwide adoption and users’ frequent engagement with them. Combining smartphone data with the wealth of information from smartwatch data [36] and real-time self-reported data through EMA can maximize the utility of digital phenotyping for prevention [46]. Despite the clinical value of this information, respecting patient privacy should remain a priority when collecting their personal data [35,37,47]. It is also important to note that accessibility to a smartwatch and a smartphone with a wireless plan is not a given for everyone. The digital divide is particularly visible among ethnic minorities, those who are older, less educated, have a lower household income, and those who have a disability [48]. However, in the near future, it can be reasonably expected that most of the world will have access to mobile devices. In the meantime, the combination of smartphone, smartwatch, and EMA data is worth exploring. Indeed, answers to EMA questionnaires enrich a passive dataset with labels, with which supervised machine learning models can be trained. Building upon the previous example, passively monitored sleep patterns can be labeled with a daily morning EMA question on subjective sleep quality.

Notwithstanding the potential of mHealth apps in preventing depression [49-51] or at least in preventing the transition from minor to severe depressive symptoms [52], engagement is low. Most of mHealth app users try an app only once [53,54]. mHealth trials endure high dropout rates, giving clues for real-life adoption problems [55]. There is also room for improvement when it comes to engagement in the long term [39]. For the population with a history of depression in particular, the lack of motivation and behavioral avoidance inherent to the mood disorder [56] might pose a major challenge to engagement and retention [57]. Furthermore, research shows that the implementation of mHealth tools in routine mental health care often does not meet expectations [58]. This is because certain prerequisites for achieving successful implementation of mHealth in practice are not met. mHealth tools need to be accepted by patients, appropriate in addressing their mental health disorder, and meet their expectations and preferences. This has thus far not always been the case. Our study closes this gap by unraveling the specific requirements and concerns of individuals remitted from depression in particular, whose opinions have been under-researched. The choice for this population goes hand in hand with the shift in mental health care from reactive to preventive approaches [59-61]. Considering the potentially far-reaching personal, social, and ethical implications this technology can have [62], user-centric design is warranted. As such, we make sure the app is acceptable, aligned with their preferences, and usable, to ensure future adoption [63].

### Objectives

Unlike previous research, which focused on preferences of individuals with current depression for interventional apps based on active data collection, this study examines the requirements and concerns of those in remission toward an early relapse detection app based on both active and passive data collection. We conducted focus groups to gauge the attitudes of individuals remitted from depression toward self-tracking. A major question was to what extent these individuals are open to the idea of monitoring early warning signs of relapse. We assessed the role technology can play in this regard. We investigated adoption intention and participants’ preferences regarding the app and its functionalities. We examined the differences between active and passive monitoring in terms of privacy and other user burdens. Thematic analysis revealed 5 main themes (ie, customization, positivity, interventions, transparency, and user burden) representing the different requirements and concerns participants voiced regarding a prevention tool, the design of which should carefully consider an equilibrium between both.

## Methods

### Reporting

To ensure transparent, explicit, and comprehensive reporting of qualitative research, we report this focus group study according to the relevant guidelines in the Standards for Reporting Qualitative Research [64] and in the Consolidated Criteria for Reporting Qualitative Research [65].

### Participants

We made use of 2 channels for recruitment. Most participants were recruited through a flyer on social media. The inclusion criteria were (1) ≥18 years, (2) Dutch speaking, (3) in possession of an Android smartphone, and (4) having experienced a depressive episode in the past, from which they had recovered at the time of this study. Prior experiences with depression were self-reported in this part of the sample, and no further clinical screening was carried out by the researchers. Other participants were recruited via email through a previous study on web-based cognitive control training for depression relapse prevention [66]. These participants had already been screened for depressive symptoms in the past. Participants were recruited until data saturation was reached, that is, until no new information was heard during focus groups. In total, 17 participants were recruited to host 3 focus groups.

### Materials

The app used in this study is currently in development by a consortium of research partners, as part of the DEDICAT (“Depression’s Digital Forecasting Tool”) project. At the time of the focus groups, the app was in the prototyping phase and was thus not yet publicly available. The app captures both active (EMA) and passive (smartphone and smartwatch data) data sources. While the active data is self-reported and subjective, the passive data is automatic and objective. They both complement each other, with active EMA data acting as labels for the passive data. In the future, these data sources will be fed into a personalized supervised machine learning model aimed at early detection of depression relapse.

### Study Design

Two days before the focus group, participants were requested to download a version of our app that only triggered the EMA questionnaires (for screenshots, see Multimedia Appendix 1). Fifteen EMA questions were selected from an EMA item repository [67] and reflect psychological constructs that are relevant to depression relapse. Examples of EMA questions include “right now, I'm feeling excited” or “since the last measurement, I have been worrying” (Multimedia Appendix 2). Participants were prompted by a notification 6 times throughout the day at random times. This sampling frequency is common in mental health research [68-75], given that mood states can change multiple times a day. Furthermore, frequent measurements are necessary to compensate for missing data, to obtain sufficient labeling for passive data, and to allow for meaningful statistical analyses. If the questionnaire was not completed within 15 minutes, a reminder notification was sent once. After another 15 minutes, the questionnaire was no longer available. The EMA data was not stored and was deleted immediately. No passive data was tracked from participants. The purpose of this preparation was merely to provide participants with an accurate experience with an active monitoring app.

Focus groups were chosen over one-to-one interviews as they facilitate dynamic interactions among participants, resulting in a broader range of ideas and insights [76-80]. Moreover, focus groups can uncover requirements and concerns that might not surface in individual interviews [76,81-83]. These advantages also hold when the topics discussed are personal and sensitive [84]. Especially in the context of the design of an app that is not yet available to the public or technology that participants might never have used before, focus group discussions can be an effective qualitative data collection method to encourage creative and abstract thinking.

Three face-to-face focus group discussions took place in the research facility in Ghent, Belgium, from February to March 2024. They were conducted by TC (MA, female, PhD candidate) as moderator, with the assistance of MM (MA, male, PhD candidate and clinical psychologist). Other than having made practical arrangements for the focus groups, there was no further contact between researchers and participants before this study. None of the participants knew TC and MM. The 3 groups counted 2, 7, and 8 participants, respectively. The number of participants in the first group was limited due to an unexpectedly high number of no-shows. The size of the group, however, did not affect the quality of the data. Reasons for cancellations across all focus groups included forgetfulness, changes in work schedules, and overrun meetings. One person canceled as they felt uncomfortable participating because they had been in a guest lecture by MM.

The focus group was audio-recorded and lasted for approximately one and a half hours. It started with an explanation of this study’s rationale and the purpose of the app. By means of a semistructured interview guide, feedback and insights were then gathered by probing into participants’ expectations, needs, requirements, concerns, and attitudes toward a depression monitoring app. The interview guide was refined after a pilot focus group hosted by TC and MM with colleagues. Example questions included “What functionalities would you like to use?” “What kind of information or feedback would you like to see in this app?” and “How would you improve this app so you would be intended to use it?”

### Ethical Considerations

This study design and informed consent form were approved by the Ghent University Department of Political and Social Sciences ethics committee (2024-05) before this study commenced. Participants read and signed an informed consent form at the beginning of the focus group. They were informed through this form that their privacy would be protected by pseudonymizing the transcripts. Afterward, they received a financial compensation of €25 (US $27.17) via bank transfer.

### Data Analysis

The focus group discussions were transcribed verbatim and pseudonymized, after which a thematic analysis was carried out by TC according to the step-by-step guide by Braun and Clarke [85]. Transcripts were coded in NVivo (Lumivero) using an inductive approach. In this approach, the researcher looks at the data from a data-driven or bottom-up perspective and identifies codes that are strongly linked to the data themselves. Codes were aggregated, split up, or discarded, resulting in a code tree that eventually translated into themes and subthemes. They were identified at the semantic level by iteratively deriving and refining the codes against the data. Semantic themes represent the more explicit or surface meanings of the data, rather than the researcher’s interpretation beyond what the participant has said.

The criteria of what counts as a theme were a combination of the importance of the theme in terms of answering the research question and the prevalence of the theme, that is, the number of focus groups it appeared in and the number of participants that brought it up. In the latter sense, themes are repeated patterns of meaning [85]. The quality of a theme also depends on its ability to overarch contradictions between participants’ preferences (see, for example, the theme of customization). The resulting themes and subthemes were discussed with MM, who comoderated the focus groups, and all other authors. Finally, the active role of the researchers’ judgment, their assumptions, and theoretical backgrounds in determining themes should be acknowledged.

## Results

### Participants’ Characteristics

The sample of 17 participants consisted of 3 men and 14 women, aged 21 to 49 (mean 27.7, SD 6.8) years. All participants were White.

### Themes

#### Overview

**Figure 1:**
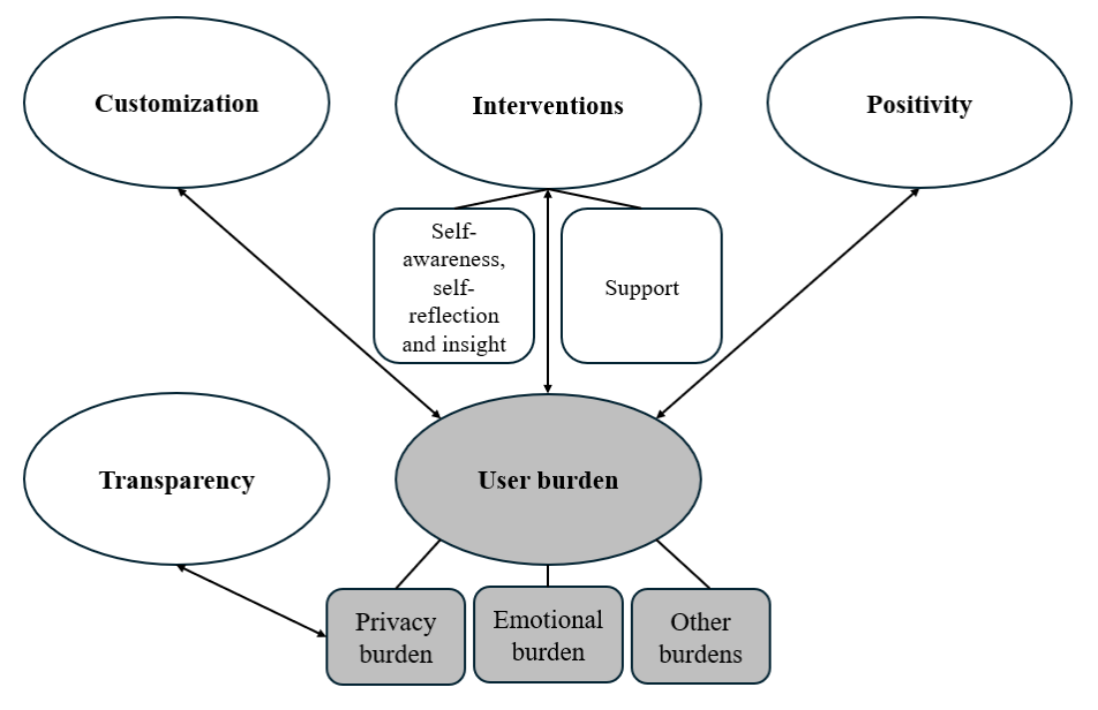
Thematic map of the 5 main themes: customization, interventions, positivity, transparency, and user burden.

The 5 main themes resulting from the thematic analysis and the relationships between them are visualized in a thematic map (Figure 1). The ovals depict the main themes, while the rounded rectangles represent the subthemes. The themes or subthemes with a white background represent what participants require in an mHealth app, whereas the themes or subthemes with a grey background depict their concerns. Many of the topics discussed within themes are interconnected with other themes. The double-headed arrows visualize which requirements balance out which concerns. Each theme is outlined below and is illustrated with a few quotes from participants (Multimedia Appendix 3).

#### Customization

The customization theme consistently appeared across all focus groups and was echoed by numerous participants. The need for customization was apparent in multiple aspects of design: (1) data and privacy issues, (2) the EMA questionnaires, and (3) the feedback functionalities.

Participants wanted to customize which data parameters are tracked by the app. Such a customization feature would allow users to disable certain parameters they deem sensitive. Furthermore, they wished to configure whether the data would be shared with their mental health care providers or not, and if so, which mental health care provider would see which data parameters at what time. Participants preferred to decide for themselves whether and when to contact their mental health care provider in case of early detection of a relapse in depression.

Another topic of customization was related to the EMA questionnaires and the possibilities for feedback (eg, on tracked mood, sleep, or physical activity). Participants wanted to configure the frequency of the questionnaires. Next to the frequency, some liked to configure the timings of the questionnaires as well, whereas others embraced the random character in which the notifications appeared. These configurations would allow users to embed the usage of the app into their daily life—for example, shifts and breaks at work—and would allow them to decide how often and when they are confronted with questions asking about their emotional state. Concerning the content of those questions, participants expressed the need to provide context by linking mood and daily life activities. This functionality would allow for more personalized feedback, which in turn would provide the user with more valuable insights. However, a considerable number of participants preferred not to see any feedback at all to prevent adverse emotional effects—demonstrating the need for customization of feedback functionalities.

#### Positivity

Participants made it clear that they wanted positivity to be included in an mHealth app and that design efforts should be directed toward establishing (1) a positive user experience, and (2) positive reinforcement and affirmation. An interesting observation here is that participants alluded to the importance of the app being more than just a relapse monitor; they saw opportunities for prevention. Participants mentioned, for example, that the app should also include (3) journaling functionalities.

With respect to a positive user experience, participants sought the ability to customize what the app looks like. The idea of choosing between a few options of themes was put forward in every focus group. Themes consist of a combination of colors, visuals, fonts, and shapes that are consistently applied throughout the app. Participants expressed explicit interest in themes with more vivid color palettes, next to the default light and dark themes, as colors were said to represent positivity. What participants meant by a positive user experience is that the app does not look clinical, dull, or depressing.

To prevent this, some participants proposed implementing positive reinforcement via gamification into the app. Gamification refers to enhancing an app with game-like elements to keep users engaged and motivated. Whereas some participants enthusiastically suggested creative ideas for gamification features, other participants thought of gamification as something inappropriate or immature that would make the app less suitable for them. Participants did, however, agree on the omission of 1 particular gamification feature: the streak. A streak is a consecutive series of actions performed without interruption. They are used to incentivize users to maintain consistent usage. Multiple anecdotes were given on how the loss of a streak in other apps caused demotivation, disengagement, or even triggered them to stop using the app altogether.

What appealed to participants in terms of gamification was the potential for positive reinforcement through game rewards, without the risk of punishment. As 1 participant (female, 31 years, ID22) put it, “we are already far too hard on ourselves,” implying that the app should reflect mildness and forgivingness. A self-monitoring app that is flexible and forgiving after noncompliance would cultivate self-compassion [86]. An example of a reward is a motivational, positive, or appreciative message after having completed an EMA questionnaire. Finishing tasks would be more rewarding when receiving such messages. Participants proposed to have the principles of positive reinforcement and affirmation implemented in the feedback functionality as well. More importantly, they wanted such encouraging messages regardless of whether the feedback indicates that the user’s mental health remains stable, improves, or deteriorates.

Other functionalities proposed by participants were related to journaling, such as a gratitude journal and the possibility to write down positive events of the day or week. What appealed to them was the ability and encouragement to reflect on positivity. This would make for a more positive user experience. In turn, a weekly feedback overview could bring back gratitude and positive events to the user and could therefore serve as a reminder of the positivity during the week. The features mentioned above were desired by participants because of their potential to facilitate a more positive outlook on life.

#### Interventions

In every focus group, participants came up with ideas for functionalities that we categorized as possible interventions that an mHealth app could offer. The content of this theme is twofold. In return for personal data and consistent efforts to comply with EMA questionnaires, participants would want both (1) self-awareness, self-reflection, and insight, and (2) support during times when their mental health deteriorates.

#### Self-Awareness, Self-Reflection, and Insight

Participants were interested in gaining self-awareness, self-reflection, and insights from their tracked data. They preferred feedback to be displayed in the form of weekly overviews of the monitored mental health parameters. It would be even more interesting to them if they were able to link their monitored mood with activities in the EMA questionnaires. This idea of adding context to mood experiences was echoed in every focus group. Participants believed that bringing back this relationship to the user in the feedback overviews could lead to the detection of patterns and help them get to know themselves. They expected the feedback to provide insight into their triggers, coping mechanisms, self-care activities, and the activities that bring them energy. Even more so, they believed it could lead to action points or motivation for behavior change, upon learning what they felt when they were doing certain activities or when they were around certain people. When asked what appeals most to participants or what would be a motivator for adoption and long-term use of the app, the main answer boiled down to the valuable insights that the app could offer. Participants stated that for weekly feedback overviews to promote self-awareness, self-reflection, and insight, the language used should not be stigmatizing. Furthermore, they required the language to be subordinate to the user’s feelings and to be carefully formulated to avoid triggering self-fulfilling prophecies. Finally, they wanted the app to be honest about the accuracy of its results and predictions.

#### Support

In addition to gaining self-awareness, self-reflection, and insight, participants expressed a need for the app to provide support during difficult times. Participants proposed a few functionalities to support them in case the machine learning model detects a decline in mental health. As the app would collect a myriad of data, participants would prefer to see this with an explanation and some examples. In their view, realizing and understanding the situation are the first steps in coping with it. Participants argued that a difficult time is often accompanied by a drop in self-reliance. Self-reliance refers to trusting and relying on one’s own capabilities, judgments, and efforts without excessive reliance on others. As self-reliance is less evident during more vulnerable periods, they saw opportunities for personalized support beyond merely follow-up. An example they gave is that the app could display personalized tips. By providing the user with their own coping mechanisms or self-care activities, the app would primarily focus on self-reliance by supporting the users’ resilience. According to participants, this advice would be empowering and would be more accessible than a visit to their mental health care provider. However, in case the problems persist, participants would like the app to advise them to contact their mental health care provider. Should they, however, feel an immediate need for a conversation in anticipation of a consultation with a professional, a help page in the app could provide an accessible way for them to contact a mental health care service. The participants argued that during visits with their mental health care provider, the feedback overviews could also act as conversation material. A few participants mentioned that the link between mood and activities is especially relevant here because it would help to overcome potential recall biases. For instance, they might struggle to accurately recall their activities from the previous week when they reported low mood in the EMA questionnaire. Related to this, as 1 participant pointed out, the EMA questionnaires could replace journals that mental health professionals ask them to keep.

Participants argued that they can recognize a relapse in themselves. However, some saw the potential of an mHealth app recognizing it in a timelier manner [57]. This early warning sign could lead them to consult their mental health care provider sooner than they would without the support of an app. Another way the interventions could prove useful in their lives—as indicated by 1 participant—is by bridging the waiting time until the visit with their mental health care provider. In the meantime, there would be something to hold on to.

#### Transparency

Participants requested transparency regarding (1) the data monitoring and (2) the explainability of the machine learning model. They required information on what data parameters would be tracked, where they would be stored, and who would be able to access the data. A better understanding was needed of why certain data parameters would be monitored and how they link to mental health. The more sensitive the data parameter, the greater their need to understand this link. If the benefit were to be explained to them, participants said they would have a less reluctant attitude. Participants also demanded transparency regarding the timeframe during which data would be monitored before they could expect any meaningful feedback.

Participants claimed that machine learning in general is nontransparent and therefore requested more explanation on how the machine learning model would work. This was said to help them in deciding whether to trust or distrust the algorithm [87]. As opposed to a lengthy and tedious mandatory read at installation of the app, participants would prefer to have explanations displayed little by little, where relevant and upon request. Progressive disclosure—where additional explanation is provided on demand—could therefore meet participants’ need for transparency in intelligent systems [88]. In case the system would detect a decline in mental health, participants would prefer to know why, in the form of an explanation, preferably with some examples unveiling which parameters are most crucial in their case. When it comes to the language used in the explanations, they would prefer the accuracy of the predictions to be communicated honestly and transparently. This way, the participants would have access to sufficient information to decide for themselves whether to trust the system and whether they can rely on the technology.

#### User Burden

The 4 main themes mentioned above can be seen as antidotes to this theme. The user burden theme contains three subthemes: (1) privacy burden, (2) emotional burden, and (3) other burdens. We classified the user burdens into subthemes according to an existing user burden scale [89].

#### Privacy Burden

A repeated observation across the focus groups was that participants raised privacy concerns. The privacy burden is defined as a system’s risk of revealing information about a user that they would prefer not to share [89]. As discussed in the customization theme, participants would prefer to configure which parameters would be tracked by the mHealth app. They also expected an option to put in personal preferences regarding whether to allow sharing data with mental health care providers. Among the most sensitive data to share, according to participants, would be location. They indicated resistance to allowing an app to track their phone usage per app category (eg, social media, gaming, communication, etc) as well, but to a lesser extent than their location. Participants considered location very personal, as it reveals a lot about one’s daily life. Furthermore, not everyone was convinced of the link between passive data (ie, smartphone and smartwatch data) and mental health monitoring. Participants viewed passive data as more burdensome in terms of privacy to have tracked compared to active data (ie, EMA questionnaires on mood), which they deemed more relevant for monitoring mental health. The initiative of a mental health care provider would, however, increase trust and credibility in the system, enhancing participants’ intention to use it [90].

Participants would expect benefits in return for tracking and sharing data to make the privacy burden fairer. In other words, they would seek a balance between the data they would “give” and what would be in it for them. Examples of benefits expected by participants were positive affirmations, customization settings, a highly personalized detection model, self-awareness, self-reflection, and insight based on personalized feedback, personalized intervention by the app in case of difficult times, and personalized help from a mental health care provider based on the tracked data.

#### Emotional Burden

Another subtheme of user burden is the emotional burden, which is defined as a system’s risk of making the user feel bad or unnecessarily worried [89]. Participants raised concerns about the potential detrimental side effects of (1) the EMA questionnaires and (2) the feedback overviews. Based on the experience that participants had gained with EMA before the focus group, they talked about the psychological influences it had on them. Even though the questionnaire was balanced in terms of measuring positive (eg, “right now, I'm feeling satisfied”) and negative affect (eg, “right now, I'm feeling down”), a lot of participants stated that the introspection for filling out the questionnaire made them feel worse than before. They personally focused primarily on the negative emotions that were asked, even after the interviewers reminded them of the fact that positive emotions were gauged as well. Indeed, individuals remitted from a depressive disorder have a stronger attentional bias toward negative adjectives (eg, worthless), compared to a never depressed comparison group [91].

Participants in the focus groups warned about the downside effect of the confrontation with these questions multiple times a day on their mental well-being. The EMA questions forced them to think about how they were feeling, while they might not otherwise have thought about their mood. This had caused some participants to start ruminating about why they were feeling a certain way. Finally, some participants indicated that the EMA questionnaires fed preoccupation with having (had) depression. Filling out the questions acted as an unwanted reminder of a challenging time in their life. They compared it to visiting their psychologist, and how they wound down these visits or stopped them altogether when their mental health started to improve again. Similarly, those participants stated that they would install and use the app in times when they feel depressed. Consequently, they would delete the app again whenever they felt better.

Participants also worried about the emotional burden of feedback that the app could display to the user. Participants were inclined to envision feedback in the more negative scenarios, for example, displaying a downward trend in mood over the last week. Confrontation with this kind of feedback could also trigger rumination and would cause participants to be worried about a downward spiral due to a self-fulfilling prophecy. The latter would occur when the user would internalize this feedback. A participant explained that feedback stating a deterioration in sleep quality could cause trouble sleeping, which might otherwise not have occurred without seeing this feedback. To prevent any potential self-fulfilling prophecies, some participants indicated that they would prefer to have the option not to see any feedback at all. Seeing their own feedback would trigger anxiety, especially if they are alone at that moment. They proposed that only their mental health care provider would be able to see the feedback and would interpret and explain it to them during a consultation. However, not everyone shared this opinion, as others would worry that their mental health care provider would confront them with their data and feedback. This discrepancy in participants’ requirements once again proves the need for customization in the design of mHealth apps.

#### Other Burdens

The last subtheme of user burden is “other burdens.” It is an umbrella term for all other practical difficulties that participants brought forward. The following types of burdens were mentioned in the focus groups: (1) time burden, (2) social burden, (3) mental burden, and (4) access burden [89]. The time burden refers to the frequent use of the system due to the EMA data collection. Many participants indicated that filling out a questionnaire daily or multiple times a day is too burdensome. It was often not compatible with their work, education, or daily life in general [57]. The social burden applies to the difficulty or inappropriateness of responding to the EMA notifications in social settings. Those notifications also distracted them from what they are doing, resulting in a mental burden. Lastly, the access burden refers to the incompatibility of filling out EMA questionnaires and wearing a smartwatch in certain jobs. For example, nurses are not allowed to wear a smartwatch [57], or some company policies prohibit employees from carrying a smartphone with them during their shift. Additionally, participants working in jobs that involve a fixed timetable (eg, a teacher) or jobs that involve consultations with people (eg, a psychologist) were not able to fill out a questionnaire at any time of the day. Many of these issues could be mitigated by customization settings that would allow the user to choose the frequency and timings of notifications.

## Discussion

### Principal Findings

This paper presents a focus group study with individuals remitted from depression on requirements and concerns related to the design of an mHealth app for early detection of depression relapse. Participants provided feedback on an mHealth app that would capture both active EMA data and passive sensing data from the user’s smartphone and smartwatch. The aim of this app in development is to feed all data into a personalized supervised machine learning model, being trained for just-in-time detection of depression relapse. In general, 5 main themes were identified by means of a thematic analysis: customization, positivity, interventions, transparency, and user burden. Whereas the first 4 themes reveal participants’ requirements of the mHealth app’s design and functionalities, the fifth and last theme covers their concerns regarding the use of such an app.

Our findings contribute to the field of mHealth and depression management in several ways. First, our unique sample consisted of individuals remitted from depression, a group at high risk of relapse but often overlooked in research and app development. Second, feedback was gathered toward a preventative tool integrating both active and passive data. Technical feasibility and scientific validation are essential, though insufficient for successful implementation in clinical practice. Considering the poor engagement with this technology thus far, mHealth apps have to be aligned with user needs as well to ensure acceptance, adoption, and usability. Therefore, our findings offer in-depth insight into the requirements and concerns of potential end users, which are discussed below.

### Requirements

First, participants emphasized the need for customization in several areas of design: data and privacy settings, the EMA questionnaires, and the feedback functionalities. Configuring what data parameters would be tracked, who they would be shared with, and when would allow participants to maintain control over their data. This would mitigate the privacy burden for them. The assumption of ownership over one’s own data and the choice of sharing it with clinicians is in line with previous studies [70,92,93]. Participants expected functionalities to customize the timing and frequency of the EMA notifications to alleviate the time, social, mental, and access burden by embedding the usage of the app seamlessly into their daily routines. This confirms the findings of many previous studies stressing users’ need for customization of the timing [92] and frequency of self-assessments [67,70,71,86,94]. The same functionalities would also address the emotional burden by allowing participants to decide how often and when they would be confronted with questions asking about their emotional state that could trigger rumination. An important remark here is that from a psychometric point of view, customization concerning timescales can lead to significantly different conclusions [95-98]. Furthermore, from the perspective of training a supervised machine learning model, sufficient EMA data (ie, a labeled dataset) is needed to generate accurate predictions. Another example given by participants of how customization could address the emotional burden was a feature to opt out of seeing one’s own feedback overviews. This was assumed to prevent adverse emotional side effects. Allowing users to select or deselect specific visualizations in a dynamic feedback interface might provide an even better solution [71]. The importance of customization settings cannot be overstated, as insufficient personalization is a perceived barrier to using mHealth apps for depression [99] and one of the main reasons for disliking the use of apps for mental health in general [90].

The second preference of participants is for the app to display and encourage positivity. As depression is associated with biased attention to negative information [100-102], participants might require positivity in an app to compensate for this information processing bias. According to them, a positive user experience can be established through colorful visuals, thereby avoiding clinical or monotonous aesthetics [71,103]. Additionally, participants suggested directing design efforts toward positive reinforcement and affirmation in the form of rewards. This can be a motivational, positive, or appreciative message after having completed an EMA questionnaire. Previous studies suggested rewarding users for their efforts with advice and compliments [92] or encouragement messages [86]. Other functionalities proposed by participants—such as gratitude journaling and the possibility to write down positive events of the day or week—are inspired by positive psychology. This field of psychology is about positive subjective experiences: well-being, contentment, and satisfaction in the past; flow and happiness in the present; and hope and optimism for the future [104]. Positive psychology interventions such as writing down good things in life or cultivating gratitude have the ability to—at least temporarily—decrease depressive symptoms [105]. Researchers provided ample empirical support in favor of positive psychology practices alleviating depressive symptoms and enhancing well-being [106-109]. Furthermore, positive psychology strategies can act as a prevention mechanism by helping to build resilience and coping capabilities that reduce relapses in the treatment of depression [110].

The third finding is that participants requested interventions to be the focal point in the app’s design. This is interesting because participants were in remission, and the researchers clearly stated that the app would aim at mere monitoring and follow-up. The importance of interventions cannot be overlooked, as it was said to be the main motivator for long-term use of the app. In exchange for accepting a certain degree of user burden, participants would expect benefits in addition to early relapse detection. Those benefits can come in the form of self-awareness, self-reflection, and insight, or support during difficult times. Linking mood with context was deemed crucial for obtaining meaningful feedback. Contextual data can be gathered through a smartwatch [111] or through EMA diaries. It is important for the latter to include (social) contextual factors next to symptoms [67,70,92,112]. Combining mood with context is key to reflecting on emotional patterns and detecting triggers to mood changes [72,113]. Participants of previous studies agreed that the act of self-monitoring in itself and the feedback based on this data can result in self-awareness, self-reflection, insight, and behavior change [57,70-73,75,86,87,92,112,113]. Increased self-awareness is expected to help in self-assessing whether they need to reach out for help from others or not [86].

During periods of mental health decline, participants would want the app to provide them with support. In line with previous studies, they would prefer the app to display personalized tips and coping strategies [86,94]. In another study, patients put forward the suggestion to have the system alert their clinician in case of a significant change in symptom levels [92]. They liked the idea of the alerts triggering personalized therapeutic advice. However, the study also highlighted clinicians’ points of view, stating that they were hesitant about the possibility of receiving alerts. Alerts would make them worry about patient safety. The clinicians were also concerned about an increased burden of responsibility, given their time constraints. A more realistic way in which the app can offer support in difficult times is by referring the user to professional help. Participants also saw value in having the feedback overviews serve as a starting point for dialogue with mental health care providers. Another way in which feedback can help, according to them, is in overcoming the recall bias, especially if significant time has passed since a prior appointment [71,86]. EMA-derived feedback can provide a more reliable overview of how patients have felt since the previous session compared to directly asking the patient. This is because the latter is often influenced by current mood [92]. Overall, feedback overviews were expected to improve communication with clinicians [57,70] and make time spent with them more efficient [72].

The fourth and last requirement from participants was transparency concerning the data monitoring and the explainability of the machine learning model. Participants viewed transparency as an antidote to the privacy burden. However, the literature is in disagreement regarding this relationship. For example, information use transparency features—as requested by participants in the focus groups—inform users about how and for what purposes the acquired information is used [114]. While many authors have speculated that said transparency features alleviate privacy concerns, this assumption has not been empirically supported [115,116]. Therefore, a dual effect is assumed. Whereas transparency as a signal of trustworthiness and fairness [117] can help to drive information disclosure [118], it can also make privacy hazards explicit and users reluctant to share sensitive information [119].

Transparency regarding the machine learning model can be addressed with explainable machine learning. By providing a transparent explanation for its results, reasoning, and predictions, this form of machine learning is made understandable for humans. Participants’ requirement for transparency is aligned with the literature. A qualitative study found that patients with bipolar disorder would prefer to see simple explanations of the data analysis behind the experience sampling feedback [70]. In another interesting study that measured stress by combining smartwatch and EMA data into a predictive algorithm, participants expressed curiosity to understand how stress can be determined [87]. More specifically, they were interested in the relationship between certain data parameters and stress. Especially those participants who articulated distrust in the stress algorithm felt the need to identify the quality and reliability of the algorithm. In contrast to the dearth of studies that did not find an empirical relationship between transparency and privacy, participants in this study viewed transparency as essential for building trust and thereby reducing the expected privacy burden.

### Concerns

Participants required customization, positivity, interventions, and transparency to bear the anticipated user burdens of mental health monitoring. The most prevalent user burdens highlighted by participants in this study were the privacy and emotional burden. Privacy concerns were related to the tracking and sharing of sensitive data. Other studies confirm that privacy concerns are among the biggest barriers to the use of apps for depression [57,99], apps for mental illness [90], and health apps in general [120]. Given that the data would be highly personal, participants require it to be safely stored to protect their privacy [67]. Among the most personal data to track and share according to participants, would be location, which is known to be sensitive to share [57,93,121]. By extension, passive data tracking instilled more privacy concerns compared to active data. This finding fits seamlessly with earlier work on willingness to participate and actual participation in passive mobile data collection [122,123]. Even though active data collection requires more effort than passive tracking, users were more willing to agree to it, as it rendered them a greater sense of control over the content [124]. By consequence, the possibility for direct control over the passive data collection process—such as an option to temporarily switch off tracking—can lighten the privacy burden [125]. What might reduce the privacy burden even more would be a functionality to specifically pick which data parameters to share and which not to ahead of time (ie, à la carte consent). This functionality is exactly what was proposed by participants in this study.

Interestingly, however, privacy issues do not necessarily translate into poor adoption, as the literature describes a discrepancy between user attitudes and actual behavior. This is called the privacy paradox [126]. Despite reporting high privacy concerns, web users disclose private information, nonetheless. Among the possible explanations of the paradox are consumers lacking sufficient information to make informed privacy decisions, and in the case of sufficient information, they are likely to trade off privacy risks for possibly gained benefits [127-129]. The latter is also known as the privacy calculus theory [130,131]. The theory holds in different contexts, including the context of mobile apps [132] and the Internet of Things environment [133]. As perceived benefits, users often expect a better personalized service [134,135]. This is where the personalization-privacy paradox comes into play, stating that users pursue personalized services, yet the collection of personal information to provide such services raises privacy concerns [114,136]. The personalization-privacy paradox is highly relevant in the mHealth context, where trust largely mediates the effects of perceived personalization and privacy concerns on adoption intention [137]. Participants in our focus groups indeed expected benefits in exchange for sharing personal information. Personalized services were on top of the list, such as a highly personalized detection model, personalized feedback providing self-awareness, self-reflection, and insight, personalized intervention during difficult times, and personalized help from a mental health care provider based on the tracked data.

In addition to privacy concerns, participants also feared the emotional burden of self-tracking. While frequent self-assessment and feedback are promising in terms of self-awareness, insights, and motivation for positive lifestyle choices, many authors also warned against emotionally adverse side effects. EMA assessments can be regarded as a continuous reminder of symptoms rather than what goes well, possibly resulting in preoccupation with mental illness [72] or symptom worsening [92]. Frequent confrontation with mood could impair the user’s adaptive coping strategies, such as seeking distraction [70]. Interestingly, emotion tracking seems more burdensome for people prone to depressive symptoms [74,138], especially because this population is known to have a greater focus on negative emotions [91]. It is, however, worth noting that participants underwent intense EMA assessments (ie, 6 times per day). While EMA assessments in themselves are predictive of depression relapse [69,139-141], they are also needed to obtain labeling for supervised machine learning. Frequent EMA assessments are therefore inevitable in the beginning stages of development of the depression relapse monitoring tool. Considering that the prediction model will be personalized, frequent EMA assessments will also be needed in the beginning for every new user to establish a baseline and labeling. For long-term usage, however, we expect a greater contribution of passive monitoring.

Participants were also apprehensive about the idea of seeing their own data and the psychological consequences of receiving said feedback. For example, some participants worried about negative feedback triggering self-fulfilling prophecies. The accuracy of feedback is also important, as suggestive or misleading feedback can lead to self-reported symptoms worsening [142]. Similar to the findings of other studies, some participants in this study therefore proposed to reduce the emotional burden by having the EMA-derived feedback explained and interpreted by their mental health professional in person [70-73,92]. The main reason for preferring not to look at their own data would be to prevent anxiety [57] and rumination [87]. Rumination is characterized by repetitive negative thoughts and is related to a deterioration in mood over time [143]. Rumination is both a symptom of depression [144] and a risk factor for the onset, maintenance, and recurrence of depressive episodes [6,145]. Given the vulnerability of potential users, mental well-being–related assessments and feedback should therefore be well considered and carefully designed to avoid harming or at least to minimize mood reactivity [87]. The emotional burden would make participants refrain from self-reporting during periods in remission, when they do not want to be occupied with their mental (ill) health [70]. This runs counter to the idea of a relapse detection app, which can only work when users start monitoring in times of remission. Contrary to the scientific evidence that follow-up is valuable in relapse prevention [146-148], many participants stated that they would only consider self-monitoring in the presence of more severe depressive symptoms.

### Recommendations

Our focus groups have identified several themes that can guide technological development. The requirements direct the attention of mHealth app developers toward the features that participants most value, whereas their concerns expose pain points for adoption and engagement. The first recommendation is to build in a wide range of customization settings for users to have a sense of agency, ownership, and autonomy regarding their mental health monitoring. Whereas the possibility to configure data tracking and sharing has the potential to counter the privacy burden, the emotional burden is tackled through customization settings regarding the frequency and timing of EMA assessments and control over how feedback is displayed. Developers can also address the latter burden by further refining passive tracking, instead of the emotionally straining active data collection. Based on the second theme, we also recommend that app developers create a positive user experience. This can be done via relatively straightforward design efforts toward colorful visuals and gratitude journaling features. We strongly discourage integrating punitive gamification features such as streaks. Instead, developers can incorporate positively reinforcing and affirmative messages to obtain appropriate user behavior. Third, it is also recommended to add functionalities that users can gain insight and support from. That includes weekly feedback overviews and presenting the user with personalized tips conditional on their mental state. The fourth recommendation for mHealth developers is to disclose transparent information about all facets of the data processing. Progressive disclosure, for example, fulfills users’ need for transparency without overwhelming them. Transparency was said to mitigate the privacy burden, for instance, by clarifying the relevance of passive data collection. Developers should also address privacy concerns by prioritizing data protection and security.

Our results also led to some recommendations for clinicians. First, as clinicians can play an important role in the follow-up of people in remission, we recommend that they continue participating in research concerning the development of this technology. Their participation could pave the way for clinical integration. They could educate patients on how the app works and how predictions are made, particularly to make them understand the relevance of passive data collection. Clinicians’ explanations and recommendations would increase patients’ trust and credibility in the system, enhancing their intention to use it. During early use or emotionally vulnerable periods, clinicians can assist patients in interpreting feedback from the app to mitigate the risk of misinterpretation, rumination, anxiety, or self-fulfilling prophecies. Clinicians are also encouraged to explore this technology’s opportunities and risks. A potential opportunity identified in this study is that EMA can act as conversation material to improve recall during talk therapy. A risk, as mentioned during the focus groups, could be that patients feel surveilled by their mental health provider. Based upon these real-world experiences, the tool could be iteratively improved.

### Limitations and Future Work

A limitation of this study is that the requirements and concerns of our sample may not be generalizable to the entire population. This is because our convenience sample lacked some diversity in terms of demographics. More specifically, most participants were relatively young, female, and White. Under-recruitment of ethnic minorities is a well-known issue in mental health research [149]. A predominantly female sample is the result of a prevalent self-selection bias in this kind of research [57,69-72,86,87,99]. This is not necessarily problematic, as females experience a higher risk of onset of depression, longer duration of episodes, and a tendency for higher risk of recurrence [150]. Women also find it easier to confide in others about psychological problems and, therefore, are more likely to seek help by attending health care services and receive diagnoses for mental health problems [151]. Females may thus form a target population likely to adopt an app that monitors depression relapse. The sample also consisted mainly of people in their twenties, which can be explained by the first recruitment method through a social media channel linked to the university. This age group is highly relevant for our research objectives because the first onset of depression is usually in adolescence, with a substantial risk of recurrence afterward [152,153]. Additionally, younger individuals are among the most prevalent users of mobile technologies and are more likely to use mHealth apps [120]. Younger individuals and females may therefore generate a wide range of ideas toward a future mHealth app for depression detection. Granted that our results are not validated in a larger and more diverse sample, we elaborated in depth on the requirements and concerns of people who have experienced depression. Despite the relevant profiles included in our convenience sample, the generalizability of the findings to more diverse populations should be tested in future work.

Another limitation is that for a large part of the sample, experiences with depression were self-reported. Research, however, indicates high accuracy in self-diagnosis for commonly occurring mental disorders, such as depression [154]. Additionally, we acknowledge that examining the requirements and concerns of only 1 group of end users (ie, individuals remitted from depression) is a limitation of this study. Future research should consider other stakeholders as well [155]. In this regard, the requirements and concerns of clinicians are of utmost importance in implementing this technology into routine practice [58]. Nonetheless, interviewing clinicians was beyond the scope of this study.

A further limitation relates to the dynamics within focus groups. While focus groups provide researchers with many advantages, there are certain drawbacks to them as well. These include the potential for dominant voices to influence the discussion [156] and the possibility for groupthink [157]. Groupthink occurs when people strive for consensus within a group, thereby setting aside personal opinions or agreeing with those of the rest of the group. The moderators aimed at preventing groupthink by providing different perspectives to the group, asking new questions, or framing questions differently [158]. The moderators also prevented outspoken participants from dominating the discussion by encouraging silent individuals to speak within the group by directly asking them for their opinion.

Finally, participants provided feedback based on a concept for an app, rather than an app they had actual experience with. Participants did interact with a prototype of the app. However, this only covered EMA functionality thus far. The remainder of their feedback was based on a hypothetical scenario for app usage (ie, active and passive mood monitoring and early detection of a potential depression relapse). In this sense, our prototype acted as a “tabula rasa” for participants to generate unbiased and diverse ideas for. Nonetheless, we also acknowledge that our findings are limited to their imagination, creativity, expectations, and perceptions. A self-evident area for future research is to evaluate the acceptability and usability of a prototype containing the depression relapse detection model that takes into account the requirements and concerns resulting from this study. Moving forward, it will also be necessary to assess whether the implementation of our findings is associated with a positive impact on adoption and engagement in a real-world mHealth context. It could, for example, be possible that customization settings induce a cognitive or mental burden, or that journaling features heighten the privacy burden. Further user testing can reveal whether expected benefits or risks will materialize when participants experience this new tool in real life. Additionally, A/B testing can empirically test the assumptions that came forward during the focus groups. For example, it can contribute to the debate in the literature on whether transparency features alleviate the privacy burden or not.

### Conclusions

This study underscores the importance of designing mHealth apps for depression relapse detection that carefully balance requirements and concerns [57]. More specifically, their design should include customization, positivity, interventions, and transparency while minimizing the privacy and emotional user burden. According to participants, active data collection was most burdensome emotionally, while passive tracking would mainly contribute to the privacy burden. Whereas externalizing tracking by moving from user-driven to more technology-driven, automated data collection has the potential to reduce the emotional burden, it would likely aggravate the privacy burden. Passive mood monitoring will only become clinically useful if patients are open to learning about its potential, tolerate it, and perceive it to be helpful. Participants required certain functionalities to be willing to bear both burdens of self-monitoring. Only then can mHealth apps start to be widely accepted, adopted, and used in clinical practice. Considering the vulnerability of the potential end users, caution is warranted in the design of mHealth apps. Therefore, collaborative efforts should persist between researchers, mHealth app developers, mental health care providers, and individuals remitted from depression. Our findings reiterate other authors’ call for designer responsibility in the mHealth landscape, especially because researchers have yet to unravel the complicated relationship between the usage of emerging technologies and mental well-being.

## Data Availability

The pseudonymized focus group transcripts are not publicly available, given the personal nature of the stories that were shared. They are, however, available from the corresponding author upon reasonable request.

## Appendix

Multimedia Appendix 1 Screenshots of the EMA functionality in the DEDICAT app. DEDICAT: Depression’s Digital Forecasting Tool; EMA: ecological momentary assessment.

Multimedia Appendix 2 Details of the EMA questionnaire.

Multimedia Appendix 3 Quotes from participants per theme.

## Abbreviations

DEDICAT: Depression’s Digital Forecasting Tool
EMA: ecological momentary assessment
mHealth: mobile health
