# Supplementary figures and images for: Requirements and Concerns of Individuals Remitted From Depression for an Early Relapse Detection mHealth App: Focus Group Study
Source: JMIR Mhealth Uhealth. 2025 Oct 23;13:e67141. doi: 10.2196/67141 (PMC12592899; doi:10.2196/67141)

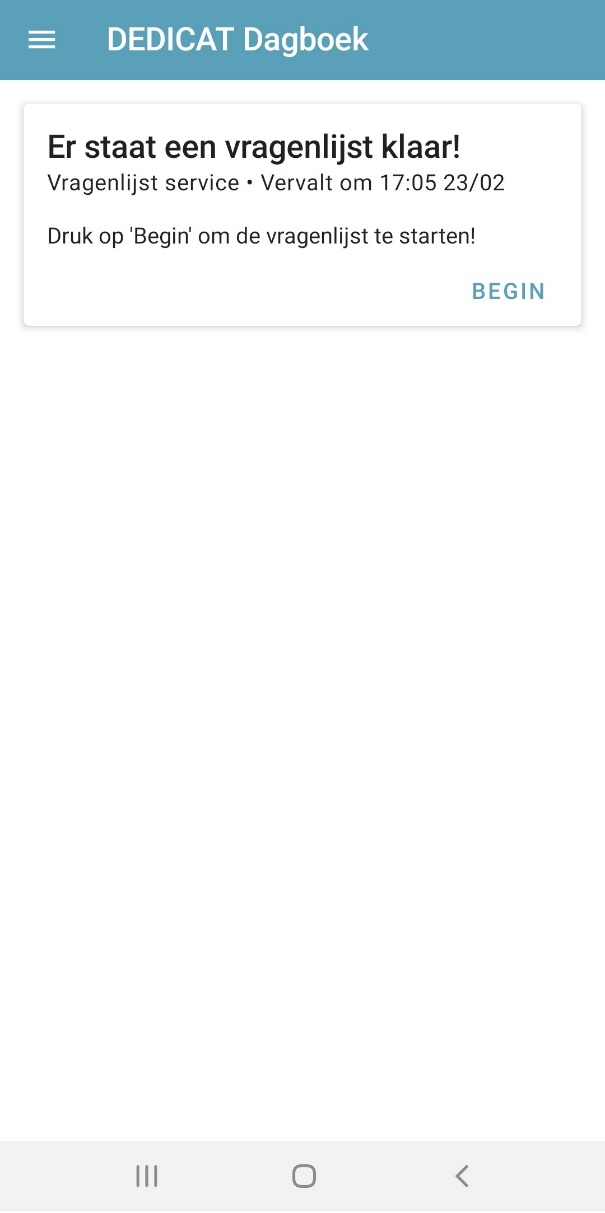

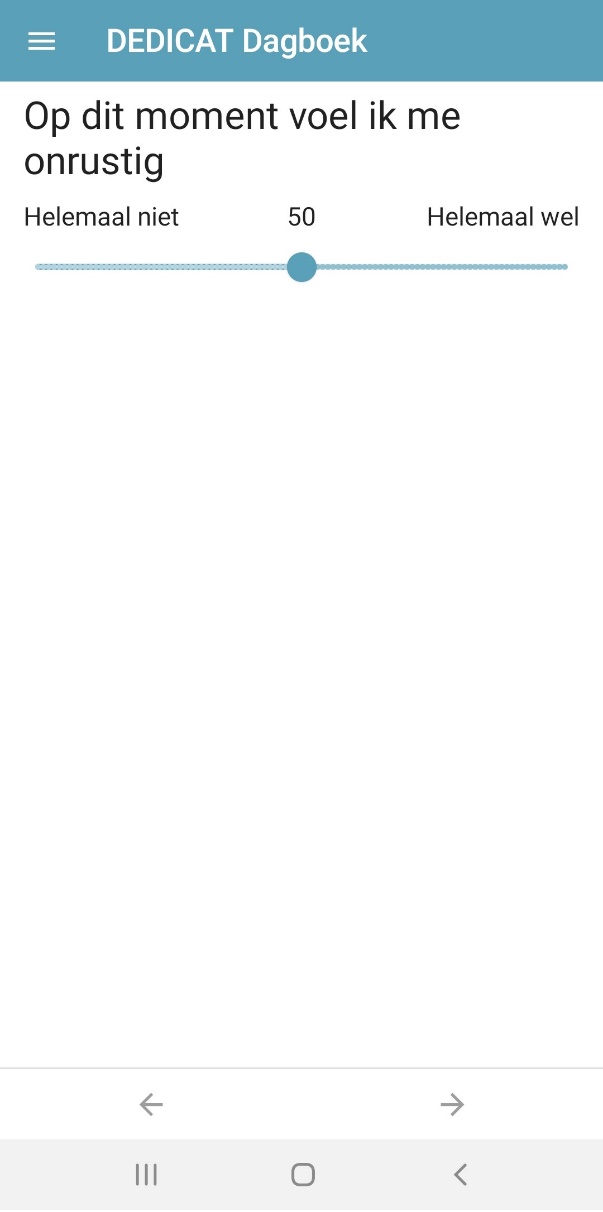

Supplement: Multimedia Appendix 1 [file mhealth_v13i1e67141_app1.docx]
