# Supplementary material for: Requirements and Concerns of Individuals Remitted From Depression for an Early Relapse Detection mHealth App: Focus Group Study
Source: JMIR Mhealth Uhealth. 2025 Oct 23;13:e67141. doi: 10.2196/67141 (PMC12592899; doi:10.2196/67141)
Supplement: Multimedia Appendix 2 [file mhealth_v13i1e67141_app2.docx]

| EMA item | Construct | Response scale | Frequency |
| --- | --- | --- | --- |
|  |  |  |  |
| The quality of my sleep was… | Sleep | VAS ^a^ 0-100 | 1 x in the morning |
| Right now, I'm feeling stressed | Stress | VAS ^a^ 0-100 | 6 x day |
| Right now, I'm feeling excited | High positive affect | VAS ^a^ 0-100 | 6 x day |
| Right now, I'm feeling down | Low negative affect | VAS ^a^ 0-100 | 6 x day |
| Right now, I'm feeling satisfied | Low positive affect | VAS ^a^ 0-100 | 6 x day |
| Right now, I'm feeling irritated | High negative affect | VAS ^a^ 0-100 | 6 x day |
| Right now, I'm feeling energized | High positive affect | VAS ^a^ 0-100 | 6 x day |
| Right now, I'm feeling restless | High negative affect | VAS ^a^ 0-100 | 6 x day |
| Right now, I feel good about myself | Self-image | VAS ^a^ 0-100 | 6 x day |
| Right now, I feel that others care about me | Connectedness | VAS ^a^ 0-100 | 6 x day |
| Right now, I feel connected to others | Connectedness | VAS ^a^ 0-100 | 6 x day |
| Right now, I am… | Alone/with others | MC ^b^: Alone, with others | 6 x day |
| Since the last measurement moment, I have been worrying | Rumination | VAS ^a^ 0-100 | 6 x day |
| Think of the most unpleasant event or activity since the last measurement moment. How unpleasant was this? | Stress reactivity | VAS ^a^ 0-100 | 6 x day |
| Think of the most enjoyable event or activity since the last measurement moment. How enjoyable was this? | Reward sensitivity | VAS ^a^ 0-100 | 6 x day |

^a^ Visual analogue scale

^b^ Multiple choice
