# Supplementary material for: Requirements and Concerns of Individuals Remitted From Depression for an Early Relapse Detection mHealth App: Focus Group Study
Source: JMIR Mhealth Uhealth. 2025 Oct 23;13:e67141. doi: 10.2196/67141 (PMC12592899; doi:10.2196/67141)
Supplement: Multimedia Appendix 3 [file mhealth_v13i1e67141_app3.docx]

### **Table S1.** Quotes from participants on the customization theme.

|  | Theme 1: Customization | |
| --- | --- | --- |
|  | Participant | Quote ^c^ |
|  |  |  |
| **Data and privacy** |  |  |
|  | Female, 21 years (ID23) | But what do you mean by mental health care providers? Because, uh, with my psychologist: yes I don't really mind that much. With my general practitioner that I only started seeing two months ago because my previous general practitioner stopped: I'd rather not. Uh. But, I think there may also be a difference, possibly, and that you could possibly tick mark yourself, what are my mental health care providers allowed to see about me. |
| **EMA and feedback** |  |  |
|  | Female, 37 years (ID20) | I’m wondering if it wouldn’t be better if you indicate which moments yourself. I’m a kindergarten teacher, you know. Like at, between 12 and half past 2 I usually have my lunch break. That's a moment for me to fill it in. You know, I can’t say to my toddlers “ah, uh, just a second, teacher ID20 is going to fill in her things”. And in the meantime, they tear down the room, so. So actually, I think beforehand maybe, yeah, that we can indicate "those are my moments". |
|  | Female, 22 years (ID6) | I also think it would be nicer if it were more personal, for example that you answer a questionnaire at the beginning of the app and that you can then put really personal things in it. Because, for example, I can get a lot out of exercising with friends, but that's every week that I can actually indicate "I feel good because – I did that". I feel good because – I did that. And that at the end you may also start to see "look, those are the reasons that you have indicated a lot in the last week: I have felt good". But that's also super personal. Whereas now it's the same questions over and over again and not necessarily questions that you connect with. |
|  | Female, 26 years (ID15) | I also think, if you get the feedback, I think maybe sometimes you're going to need that and other times you don’t. Some people are going to want to know that, others hm, maybe are more like in the moment I fill in the questionnaire, and that's enough for me and don't want, yeah, the big picture or whatever, really want to focus on that moment. So, I think maybe it should just be able to make an option like: I want to see it or I don't want to see it. |

^c^ Quotes have been translated from Dutch and paraphrased where necessary to improve comprehension

### Table S2: Quotes from participants on the positivity theme

|  | Theme 2: Positivity | |
| --- | --- | --- |
|  | Participant | Quote ^c^ |
|  |  |  |
| **Positive user experience** |  |  |
|  | Female, 25 years (ID4) | I would work with some color, because otherwise it would be very gloomy (laughs). |
| **Positive reinforcement and affirmation** |  |  |
|  | Female, 26 years (ID15)  Female, 28 years (ID18)  Female, 31 years (ID22) | For me, it doesn't necessarily have to be with a kitten and all that (referring to gamification). For me, it can be a bit sleeker and (laughs) yeah. You have people for whom that's going to work better, but I'm more of a person that likes it short and to the point. And, uh, for example, uh, “you did a good job”, uh, or “nice that you filled in the questionnaire”, but with a kitten and coins, that might be a bit too far for me (laughs).  Rather a positive message, if you filled it out, that you will then see a positive message of yeah, something, yeah.  Positive affirmation. |
|  | Female, 21 years (ID23) | I also just think, it's not necessarily because you're having a hard time that you're not doing well. I think that to keep going – despite the fact that things are not going well – that that is also, well, very strong. And we were also talking just now about those positive affirmations and I also find that even with negative results, for example, so to speak, there can be a, yeah, not necessarily a positive message of “everything will be all right” because that is very easy to say, but still a “you are doing well” or “you are trying your best” and, yeah. In any case, it's actually positive after all that you're doing that. So that in itself is just positive. |
| **Journaling** |  |  |
|  | Male, 29 years (ID14)  Interviewer  Male, 29 years (ID14) | What I may be thinking is like, but I know for example that helps for me, but also for example with other people that I know, is for example at the end of the week saying "what did you really like about this week or what are you grateful for". You don't have to do anything with that data, if you just say "I really enjoyed going to the movies or something". Then at least at the end of the app you’re just already going to feel positive by filling that in.  So looking back on your week: what was fun?  Yes, because then you had that question for example “did something fun happen” and then I say “yes”, hup, next question. But actually I just want to fill that in, even if you don't do anything with it. Because then you just really think more about the positive that you still have, that is still there in a week. |
|  | Female, 22 years (ID6) | I also think when things are going badly for you, that's really something to look back on. That you see, in the last two months, every day you have written down something that you are grateful for, look back at that and you might realize it more. |

^c^ Quotes have been translated from Dutch and paraphrased where necessary to improve comprehension

### Table S3: Quotes from participants on the interventions theme

|  | Theme 3: Interventions | |
| --- | --- | --- |
|  | Participant | Quote ^c^ |
|  |  |  |
| **Self-awareness, self-reflection and insight** |  |  |
|  | Female, 21 years (ID23) | But I would also like it and I don't know if that's the plan: to see the data itself. Uh, because that's very, I really like to track data, when I keep track of something like that and then I see it all nicely in a graph and then I'm like "YES". Uh, and yeah, I think that could also be fun to effectively, yeah, see your data and also look for yourself. You know, I'm filling in a diary now because then I can look back and think "oh right, I did feel good then", while I think I've been depressed all week. That's not true. I do have good days, and well, seeing that data, you know, might be pleasant as well. |
|  | Male, 25 years (ID3)  Female, 23 years (ID2) | I think it's very important for the app to, uh, phrase it as “are you feeling this way?” rather than saying “this was your week now, you are feeling this way”. And that they don't immediately come off with a stamp, but that they say like “we notice a few things, could it be that you are feeling down this week?”. You see, that you really just don't get a stamp on it, because that's not pleasant.  Yes, because otherwise you get such a situation when people tell you “ah, you're angry huh?”, but then you're not angry, but then you end up getting angry because people call you angry. You know. |
|  | Female, 22 years (ID6) | Such a kind of activities that you can really link that to moments that at the end you can look back on and see “this helps me tremendously”, “these are moments that I, that I very, that can pull me down very hard”. And that you really personally, that you can personally link it to that because sometimes you don't realize that, that certain events or people make you unhappy. And those can also be things that you can take to your psychologist and then really have personal help. |
|  | Male, 29 years (ID14)  Interviewer  Male, 29 years (ID14) | Yes, I also think, that can effectively also help a little bit positively with even a relapse, to hold it back again. Because with a diary you can effectively, if you fill it in, if you do that for 2 weeks it does help you to look at things a little more positively or to estimate for yourself “do I need help?”.  In what ways could such questionnaires help you?  Because still, yes, now they were, now I thought they were a little ‘too’ maybe, a little too simple, or in a certain way, but still to think about “okay, what has really stressed me out this week?” and “can I do something about it?”. If, for example, that's something you can fix, and think “okay, maybe I can adjust the situation”. And, for example, in my previous job, that could help prevent a burnout, for example. I mean, that would be really useful to think about for yourself. |
| **Support** |  |  |
|  | Female, 25 years (ID4) | But then I thought maybe about the fact how it works now with the questions. If from that a kind of short conclusion or so can be drawn like "Look, we notice that you're having a bad day today” or something, and that from there tips or so come or something like that. Perhaps we can also indicate in advance “what are things that help you”, so that this can be taken into account and implemented, so that if it is noticed that “today is not going well - look, these are things that help you”, that this can be brought back or so that it also becomes more visible for yourself. Because at a moment when you are really not feeling well, it is difficult to come to something like “okay, this is what I really need or this can really help me and it doesn't really take that much effort or time”. |
|  | Female, 24 years (ID5) | Hm, I think that's also the strength of such a mobile app then. That you can keep track of, ah yes, that's how I'm doing and that you can then say - because it's very difficult when you're in it, to realize that I'm actually not doing so well. And if you can objectively see in there “indeed, these are the signs that things are actually starting to go downhill for me”. And that as a result you then take that step again, like “okay, now I need that help again”. Because exactly, I was also like “yeah, now I really don't need to go to the psychologist very frequently anymore”. Indeed, every now and then, I also did one of those maintenance sessions (laughs). Sounds so short-sighted now, but... Uh, but that you can also see something “yeah, okay, and now I need it back”. Because sometimes it takes a very long time before you realize yourself “ooh, it's actually going downhill again, now I need to go back”. And then you almost have to hit the wall by the time it gets to that point again. |

^c^ Quotes have been translated from Dutch and paraphrased where necessary to improve comprehension

### Table S4: Quotes from participants on the transparency theme

|  | Theme 4: Transparency | |
| --- | --- | --- |
|  | Participant | Quote ^c^ |
|  |  |  |
| **Data** |  |  |
|  | Female, 25 years (ID4) | I guess I would just like to know “who does it end up with and what is actually done with it”. |
|  | Female, 23 years (ID9) | But I do think what was said: if you know "it's going to be 3 to 4 weeks", that you can indeed adjust to that and that that will go better than if you just don't know for 3 to 4 weeks when something is going to come. So if you just know it very well beforehand "this is what is going to happen and from then on you're going to get feedback from that", that that will help. |
| **Explainable artificial intelligence** |  |  |
|  | Female, 22 years (ID13) | But I also have that in general with that artificial intelligence that I'm also like "hm yeah, because as you say you don't really ... yes it's not really clear or something". |
|  | Female, 22 years (ID13)  Female, 25 years (ID4) | Personally, I would like to have an explanation. Yes.  Yes, I think so too. I also think it would be interesting to have an icon or something like that next to the things that happen, where you can see the explanation, so that you can decide for yourself "I want to know more about it or not". That it's not necessarily a very long list of explanations at the start of the app that nobody completely goes through I think. |
|  | Female, 31 years (ID11) | It depends on how it is displayed. If it's really short: no. I would then want to see that with an explanation like “look, we see that this is because you, you already have a lot, that your stress, well, your heart then beat a lot, you've also indicated many times that you feel gloomy”. So with a number of examples that I think “yes, I indeed did that”. And being kneaded into that realization but not just “BAM”. That wouldn't work. |
|  | Male, 29 years (ID14)  Interviewer  Male, 29 years (ID14) | I'm pretty skeptical about that. Uh, I think just like artificial intelligence, I do use that. But if it says something wrong once, then I'm immediately like “okay, from now on the trust is gone and I'm always going to question it”. But for example with such a watch, if it once says “you're stressed” and I'm not, I'm going to immediately say “yeah, I'm taking you off”, because you know, I mean, I can't trust you huh. I mean, with other machines, if you want coffee and no coffee comes out, you're also going to be like “yeah, that one is broken”. So, anyway, I think, and then with such an app, for me, by the time it can really work, it has to be very accurate. But I also know, doctors and psychologists are not always accurate either, but then you have to be honest. Then you do have to say “chances are that...”. Because really, 100% certainty or 75% certainty, then I think like “okay, what is that based on, why not 76, what is your data?”. I find that very... But that's actually, I'm very skeptical about that.  In which way would you prefer to see that?  Yes, because otherwise... That it still sees itself as a subordinate thing to your own feelings. Uh, like I just said “who do you believe more then, the app or yourself?”. From the moment you start believing it more, while it's not 100% accurate, I also don't think it's that useful that you really, that you then indeed say: “now I have to do this from the app, so I'm going to do it, now I'm going to do this”. I find that, I would find that a shame. |

^c^ Quotes have been translated from Dutch and paraphrased where necessary to improve comprehension

### Table S5: Quotes from participants on the user burden theme

|  | Theme 5: User Burden | |
| --- | --- | --- |
|  | Participant | Quote ^c^ |
|  |  |  |
| **Privacy Burden** |  |  |
|  | Female, 23 years (ID9) | Hm, if I may perhaps think very practically with the things listed there as well. I personally would not approve of my GPS location and apps. That would be a very big barrier for me to effectively install that app or not. So that you can choose whether you have that tracked or not. From a purely practical point of view, I would try to put that in there though. |
|  | Male, 29 years (ID14) | Maybe the mobile phone already knows a lot about me. Uh, but yeah, I still think that it's going to know a little less than effectively that active data. |
|  | Female, 22 years (ID6) | I would also feel very watched and, well, afraid that – even if things don't go badly – that I would be a bit afraid that too many people, yeah, have control over that. While, not necessarily hacked or anything, but also that I think "I might be on my mobile phone a lot for a few days now". I don't necessarily want my psychologist or anyone to know that, because it's not necessarily a bad thing. So that's why I would also be a bit scared, I would feel very watched if all that information would be shared. |
|  | Male, 25 years (ID3)  Female, 23 years (ID2) | Now, the notifications that you get, I think if you could set the GPS to a home location or something, that those notifications are sent when you are at home, that would be more interesting. Because if you're on the road and you miss, yeah, you miss the survey or you're in class or at work or something like that... Yeah, then you don't really have the time or feel like filling in a questionnaire like that.  Then that does seem like a functional benefit to me indeed. If I know that this is a functionality, I would be more inclined to give permission for that location because it offers a functional advantage. Because other than that, I don't really see the relevance - I must say – you know, unless, of course, okay, if you stay at home for two weeks, then indeed - as someone said there - I understand that that is an indicator. But other than that, whether that I'm now, or that that, that thing knows whether I'm in the supermarket now or on holiday in England or sitting at home, what difference that makes exactly, that's not entirely clear to me. |
| **Emotional Burden** |  |  |
|  | Female, 27 years (ID1)  Interviewer  Female, 27 years (ID1)  Interviewer  Female, 27 years (ID1) | But if the questions would be more neutral, that would already be something completely different I guess.  More neutral, how?  Yeah, because now it said: are you down, or, or, are you irritated. And indeed those feelings are so negative, uh....  Of course, it also said excited and energized.  Oh yeah, that's right...I'm more focused on the negative, yes it's true. |
|  | Female, 27 years (ID1) | This morning I felt very cheerful and energetic and then I was all "yes, energetic, yes, cheerful, yes" (laughs). And then in the afternoon I was like, yeah I don't know, a bit less so, and well, just before I was on my way over here I had to fill it in again and I had to fill in more negative things. And I did think "ah, gee, do I feel less today?". You know, indeed, that’s something like "oh oops yeah, I feel less today". If I wouldn’t have been confronted with that, I wasn't going to question it much. Yeah, you know, it is confronting in that sense. Because you, you start to think "Hm, but why do I feel less? And maybe it’s because of this or that". And, you know, that brooding, that may indeed be something typical for people who have had a bad period because you're overthinking a lot, so yeah, I do recognize what she says. |
|  | Male, 25 years (ID3) | Yeah, I see, you know, I think it's a very noble app, but I don't really see why you would install it when you're in a period where you're just coming out of a dip, that you're actually feeling good about yourself. And that you think "I'm going to install that because I want to be reminded of that every now and then and just see that I don't relapse". But if I was feeling super good or really good about myself and good in my life, I wouldn't be like "oh, I'm going to download this now just to be sure". |
|  | Male, 29 years (ID14)  Interviewer  Male, 29 years (ID14)  Female, 31 years (ID22)  Male, 29 years (ID14) | But then I am indeed thinking more about how general practitioner visits work. For example, if you’ve had blood drawn, you can actually already see that online, but it’s still better to sit down with the doctor and have him explain it, like "okay, this means such and such". Because otherwise you're going to fill it in all by yourself and interpret those graphs like "it's so bad and it’s always going down and". Then I see, well, I know for me: data is great, but if data goes down, no matter what it is, your feelings, your finances, doesn't matter, if you see a constant line. You know, that's so, I wouldn't go well on that. I would prefer that your psychologist has that data, that I don't know it myself, that we visit, that she can then frame that as "okay, it's so and so and so, I've noticed that, why could that be". And so on.  So you wouldn’t want to see your own data at all?  I would like to know from my psychologist or general practitioner, but interpreting it myself, then you go on the internet to search even further and then you know, I mean...  Anxiety arises (laughs).  Yeah, and if you're sitting alone at that moment, then pff, yeah. |
|  | Female, 22 years (ID6) | I would also be very afraid to be confronted with that or something. If you then go to your psychologist, to be told "yes but you did this or you were at home for two weeks". |
| **Other Burdens** |  |  |
|  | Female, 26 years (ID15) | So for me to fill in something every day, that would already, that would already be too much actually, yes. |
|  | Female, 22 years (ID13) | Yes, or maybe, I tended to put it off or not do it because you're with others and it's, for me that's a barrier to say "okay, yes". You know, that's similar to a situation like the one we are in right now. You're in a focus group, you're in class, you're with friends and then you have to take out your mobile phone, fill out that questionnaire. You know, it would also be weird if I get that questionnaire now and say "yeah, guys, 2 minutes, I have to fill this out here". |
|  | Female, 23 years (ID2) | For me, because I, um, I know one of the reasons why I used to feel really bad was a huge obsession with my phone and all the time being busy with "have people looked at all my messages, have they replied to them, have they..." and so on. Stuff like that. And that's exactly why – since I've recovered from that – I've come into a very conscious way of thinking in which I'm not busy with my phone when I'm busy with another, when I'm busy with something else. For example, I'm cooking, I'm not looking at my phone. I'm uh, that watch also helps with that because then I see when something very important comes in, then I know "ok, I have to look at my phone". But other than that, I see "all those notifications aren't important, I don't have to do anything with my phone". And that's exactly why I think if I get a notification like that and I have to fill it in within fifteen minutes and I'm busy with something else, then I'd be annoyed by that because that takes me out of my, what I'm doing. |
|  | Female, 31 years (ID11) | But for me, for example, yes, at work, my mobile phone is just in my bag and I don't look at it for the entire day, except once during my lunch break and in the evening before I leave and maybe in the morning. But other than that, I'm not going to have seen all those notifications, simply because that's not possible in my job. So for me, yeah, I can't suddenly say “I'm going to fill that out now”. There is no room for that. So, if you then say 2 or 3 times, I would then indeed do that in the morning, afternoon and evening or so. Simply because otherwise, there is no room there, personally for me. |

^c^ Quotes have been translated from Dutch and paraphrased where necessary to improve comprehension
